# Uncertainty in Determination of Meteorological Drought Zones Based on Standardized Precipitation Index in the Territory of Poland

**DOI:** 10.3390/ijerph192315797

**Published:** 2022-11-27

**Authors:** Joanna Wicher-Dysarz, Tomasz Dysarz, Joanna Jaskuła

**Affiliations:** 1 Department of Hydraulic and Sanitary Engineering, Faculty of Environmental Engineering and Mechanical Engineering, Poznan’ University of Life Sciences, Piątkowska St. 94A, 60-649 Poznan, Poland; 2 Department of Land Improvement, Environmental Development and Spatial Management, Faculty of Environmental Engineering and Mechanical Engineering, Poznan’ University of Life Sciences, Piątkowska St. 94E, 60-649 Poznan, Poland

**Keywords:** meteorological drought analysis, standardized precipitation index (SPI), spatial interpolation, Python scripting

## Abstract

The primary aim of this work is to assess the accuracy of the methods for spatial interpolation applied for the reconstruction of the spatial distribution of the Standardized Precipitation Index (SPI). The one-month version called SPI-1 is chosen for this purpose due to the known greatest variability of this index in comparison with its other versions. The analysis has been made for the territory of the entire country of Poland. At the same time the uncertainty related to the application of such computational procedures is determined based on qualitative and quantitative measures. The public data of two kinds are applied: (1) measurements of precipitation and (2) the locations of the meteorological stations in Poland. The analysis has been made for the period 1990–2020. However, all available observations since 1950 have been implemented. The number of available meteorological stations has decreased over the analyzed period. In January 1990 there were over one thousand stations making observations. In the end of the period of the study, the number of stations was below six hundred. Obviously, the temporal scarcity of data had an impact on the obtained results. The main tools applied were ArcGIS supported with Python scripting, including generally used modules and procedures dedicated to geoprocessing. Such an approach appeared crucial for the effective processing of the large number of data available. It also guaranteed the accuracy of the produced results and brought about drought maps based on SPI-1. The methods tested included: Inverse Distance Weighted, Natural Neighbor, Linear, Kriging, and Spline. The presented results prove that all the procedures are inaccurate and uncertain, but some of them provide satisfactory results. The worst method seems to be the interpolation based on Spline functions. The practical aspects related to the implementation of the methods led to removal of the Linear and Kriging interpolations from further use. Hence, Inverse Distance Weighted, as well as Natural Neighbor, seem to be well suited for this problem.

## 1. Introduction

In recent years, climate change has become the most important problem in the world due to increasing temperatures and extreme climatological processes including droughts, fires, and floods [1]. Spinoni et al. [2] have reported that droughts during the 20th century occurred on a global and regional scale. The Sixth Assessment Report of the Intergovernmental Panel on Climate Change (IPCC) predicted that the global mean temperature will be increasing every year in the future by 1.1 °C compared with the period 1850–1990. Due to Guo et al. [3], the frequency of extreme conditions has increased since 1950 and has depended on the geographical area including land use, form, and climate parameters. Recently, Poland has been one of the regions most seriously affected by climate warming. Droughts occur frequently, and water resources are seriously threatened [4]. Karamuz et al. [5] evidenced the increasing occurrence of drought with varying intensity over many regions in Poland in the last years. Although there are many papers presenting the changes in temperature and precipitation on a regional scales, there has been no assessment of the spatial extent of drought events in the total area of Poland.

Drought can be defined as meteorological, agricultural, hydrological, and socio-economic, depending on the number of days below a certain rainfall threshold, soil moisture deficiency, and level of surface- and groundwater. According to Peters-Lidard et al. [6], all types of droughts influence and transform each other and there is a strong correlation between different types of droughts. A meteorological drought occurs when dry days dominate in a particular region, mainly connected to atmospheric changes [4]. Hydrological drought is defined as low water levels and flows in rivers, lakes, and groundwater, which usually depend on meteorological drought [7]. In turn, agricultural drought occurs when climate change affects crop yield [8]. Socio-economic drought occurs when water resources systems could not fulfill the water demand and have an impact on the economy [9].

Droughts affect water resources, management and planning strategies in catchments. The identification of the frequency and spatial extent of drought, expressed in a number of specific indices, helps in risk management studies. Drought indices are calculated using databases of precipitation, soil moisture, crop yield, flows, and surface water and groundwater levels. Previous studies used Standardized Precipitation Evapotranspiration Index SPEI [10], Palmer Drought Severity Index PDSI [11], Modified Palmer Drought Severity Index MPDSI [12], Standardized Precipitation Requirement Index SPRI [13], Surface Water Supply Index SWSI [14], Standardized Soil Moisture Index SSMI [15], Standardized Runoff Index SRI [16], Standardized Water Supply and Demand Index SWSDI [17], Normalized Difference Vegetation Index NDVI [18] and Streamflow Drought Index SDI [19].

The Standardized Precipitation Index (SPI) was recommended by World Meteorological Organization to assess the frequency, intensity, and duration of droughts. SPI has been used in meteorology, agriculture, and water resources by researchers to analyze droughts in different geographical regions because its calculation requires only the precipitation data [20,21,22,23,24,25,26]. Additionally, SPI allows the calculation of changes for any time and spatial scale. However, a serious limitation of the SPI calculation is the high sensitivity to the length of the period over which the statistical data were collected [22].

The aim of the study is to analyze the accuracy and uncertainty of the methods for spatial interpolation applied to the reconstruction of the spatial distribution of SPI in the territory of the entire country of Poland. Different versions of SPI are used for the analysis of precipitation anomalies and water resources scarcity. The main classification of these indexes is based on the temporal resolution, which is included in the name of the index by the addition of the number of months to the index name. Hence, SPI-1 is the index calculated with one-month sums of precipitation, while SPI-6 is applied for six month periods. Due to the seasonal variability of the precipitation, longer periods should be preferred for the assessment of the water resources availability, e.g., SPI-6, SPI-12, etc. However, the SPI calculated for shorter periods is more variable and it seems to be more suited for the assessment of uncertainty in determination of drought extents. This is the reason why SPI-1 is the basis of the presented analyses. The analysis has been made on the basis of the publicly available data. The variability of SPI-1 was assessed for the period 1990–2020. However, all data available for the period of observations 1950–2022 were implemented for determination of this index. Two kinds of basic data were used: (1) measurements of precipitation and (2) the locations of the meteorological stations in Poland. The number of available measurement stations varied in the analyzed period. In the beginning, in January 1990, the number of available stations was over one thousand. At the end of the period, there were fewer than six hundred stations. This temporally dependent scarcity of data was also crucial for the obtained results.

As the basis for the investigations, ArcGIS and Python scripting are chosen. This combination was crucial for the effective and accurate processing of a large number of data and the efficient generation of drought maps based on SPI-1. It also opened some new opportunities for the future development of the presented methodology. The five spatial interpolation methods were tested: Inverse Distance Weighted, Natural Neighbor, Linear, Kriging, and Spline. The availability of the algorithm and tools in the ArcGIS toolboxes determined the choice of the methods, which is explained further. In general, the results of the presented research question the common recommendation of SPI interpolation with the first of the mentioned methods. It seems to be possible to effectively apply other methods, too. Theoretically it may happen that the rest of the listed procedures provide better results than the Inverse Distance Weighted. The research presented should answer this question and clarify all doubts in this area.

## 2. Climatic Conditions in Poland

**Figure 1:**
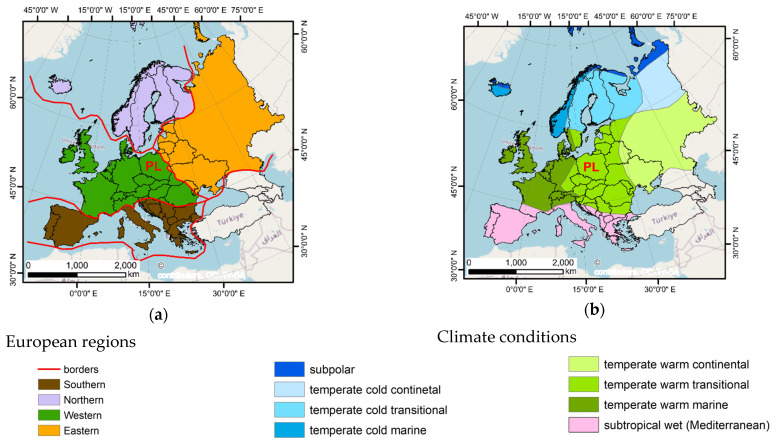
Regions and climatic conditions in Europe: (**a**) map of regions according to the physical and climatic conditions, (**b**) types of climate conditions, PL = Poland (based on Wiking-Educational Publisher, license: CC BY 3.0).

Poland is located in the central part of Europe, which in many cases is specified as a region located between the west and the east of Europe. This specification is reasonable when political and economic circumstances are considered. However, the classification of regions according to the physical and climatic conditions does not include such a specific area. For example, Kondracki [27] suggests that the major part of Poland is located in Western Europe. Some very small part of the country is in Eastern Europe (Figure 1a).

According to the approach introduced by the above-mentioned author [27], there are three large Western European megaregions, which split the area of Poland. Between them, the region of Central Europe out of the Alps and the region of the Carpathian Mountains may be mentioned.

There is also the eastern European Lowland, which is a part of Eastern Europe [27]. The climate conditions are formed by the location in moderate latitudes, between 49° and 55° of the northern hemisphere.

Such a geographic position is responsible for the fact that Poland is located in the zone of the temperate and transitional climate (Figure 1b). The climate in Poland depends on the Atlantic Ocean in the west and the land of the Euro-Asia in the east. An important factor is also the distance from the Baltic Sea [28]. The climate of the temperate latitudes of Poland is characterized by large seasonal variability. This is why the winters in Poland very often are frosty but may also be moderate and long. On the other hand, the summers can be rainy and hot [28].

Such conditions are formed by the distribution of temperatures as well as the amount of precipitation in the country. The maximum annual precipitation is observed in the mountains in the southern part of the country. The range of precipitation variability there is from 700 mm to 1800 mm. The greatest values are measured in the meteorological stations located at the tops of the mountains. The smallest amounts of annual precipitation are observed in the lowland in the central parts of Poland, especially in the regions of Wielkopolska and Kujawy. The annual precipitation there may vary from 500 mm to 600 mm. In extreme conditions, 400 mm may be also observed [29]. The mean temperature of the air in Poland during the analyzed period 1990–2020 was 8.73 °C. However, this parameter in the last 10 years (2011–2020) was greater and equaled 9.33 °C [29]. On this basis, the authors of the report “Climate of Poland 2020” [29] have assessed that the hottest year observed since the middle of the 20th century was 2019, when the mean temperature of the air in Poland was 10.2 °C.

## 3. Materials and Methods

The period considered covers the years 1990–2020. Two kinds of basic data are used: (1) measurements of precipitation, and (2) the locations of the meteorological stations in Poland. The measurement data are publicly available and may be downloaded from the official web page of the Institute of Meteorology and Water Management in Poland (IMGW = Pol. Instytut Meteorologii i Gospodarki Wodnej) [30]. Monthly sums of precipitation verified by the publishing institution were used. All the available observations, since 1950, were downloaded and used for the calculation of the necessary statistics.

The location of the stations may be found in the IMGW-Hydro [31]. It is visible that the density of stations also varies in space. The number of stations is greater in the southern part of Poland, where the observed magnitudes of precipitation are higher.

**Figure 2:**
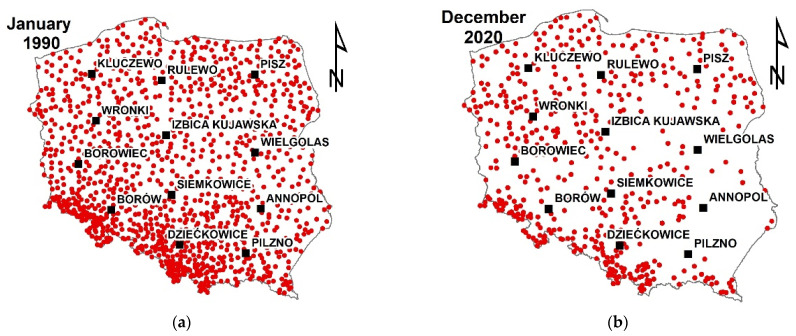
Density of the meteorological stations in the analyzed period: (**a**) in 1990, (**b**) in 2002.

The number of available measurement stations also varied in time during the analyzed period (Figure 2a,b). In the beginning, in January 1990, the number of available stations was over one thousand. In the end, there were fewer than six hundred stations. The obvious reason for such a change was the reduction of cost related to the functioning of the meteorological network in Poland. However, it is worth mentioning that the stations are modernized and provide higher-quality results.

The other data used for the presentation of the analyzed concepts include measurements of temperature all over the country and the borders of the country. The first are also publicly available and may be downloaded from the same locations at which the precipitation measurements were made. The boundaries of the country were taken from General Bureau of Geodesy and Cartography (GUGiK = Pol. Generalny Urząd Geodezji i Kartografii) [32].

The Standardized Precipitation Index (SPI) is a well-known indicator of precipitation anomalies, e.g., [33,34,35,36,37,38]. It is especially useful for the detection of water scarcity and drought conditions. The European Union institutions [39] as well as Polish officials recommend this indicator for the assessment of the atmospheric drought. The presented research refers to the document released by the Polish National Board for Water Management (KZGW = Pol. Krajowy Zarząd Gospodarki Wodnej) [36]. The procedure for the calculation of SPI consists of two steps: (1) normalization of precipitation measurements, and (2) calculation of the index. The first step may be carried out with different formulae [34]. However, the quoted document by the National Board for Water Management recommends the use of the cubic root of precipitation

(1) $$U=\sqrt[{3}]{P}\;,\;$$

where *P* is measured sum of precipitation and *U* is a transformed sum of precipitation. In general, the sum of precipitation *P* as many natural environmental variables is well described with the gamma distribution, which is not very convenient for further analysis. However, many studies by Polish researchers on Polish data of the same type have proved that the transformed sum of precipitation *U* is normally distributed e.g., [34,36]. The values of *U* are calculated based on monthly or longer sums of precipitation. Then SPI may be calculated according to the following formula

(2) $$\mathrm{SPI}=\frac{U-\mu }{\sigma },$$

where *µ* is the mean of *U* calculated over the analyzed period and *σ* is the standard deviation. Such an index is negative when the precipitation is lower than the mean, and positive otherwise. Its values measure the distance of the current precipitation conditions from the mean conditions. Because *U* is normally distributed, the SPI may be classified as suggested in the quoted document and presented in Table 1. The recommended color denotations are also presented in this table.

Because the main aim of this study is the analysis of differences between the methods of interpolation, the most variable version of SPI was taken into account namely SPI-1. The values of SPI-1 are determined on the basis of monthly precipitation sums.

The main tool applied in the presented research is the ArcGIS software developed by the ESRI company. In particular, the tools available in the extensions, such as Spatial Analyst, 3D Analyst, and Data Management, were applied [40]. The most important are algorithms of the Interpolation toolset, which include the methods of interpolation. Five methods were tested: (1) Inverse Distance Weighted, (2) Natural Neighbor, (3) linear method, (4) Kriging method, and (5) Spline method. Only the tools representing pure mathematical methods were tested. The algorithms consisting of combined mechanisms or mixtures of the methods were not analyzed. Hence, such tools as “Topo to Raster” were not considered.

The first of the tested methods, the Inverse Distance Weighted (IDW) one, is recommended for the spatial reconstruction of SPI zones. It is also a well-known method in the environmental sciences. In the algorithm of IDW, the interpolated value in a particular point is calculated as a weighted sum of neighboring points, where the inverse distances play the roles of the weights. Such a mechanism guarantees the continuity of the interpolation surface. If the point of interest where the particular value is interpolated is located between the sample points with the assigned values, the IDW interpolation should not overestimate or underestimate the interpolated values [40,41,42].

The internal mechanism of the Natural Neighbor method is different. It is based on Voronoi (Thiessen) polygons associated with the points of samples surrounding the point of interest. The main idea is to create a new polygon assigned to the point of interest and to use the areas cut from the previous tessellation as weights. The method is very simple to use, because it does not require any initial data, setting of parameters, etc. It does not also overestimate or underestimate values. The interpolated value is always in the range of the surrounding values [40,43,44].

Although linear interpolation is not explicitly available in ArcGIS, it may be easily emulated with the tools available in this software. The equivalent of this method is the algorithm composed from the creation of a Triangular Irregular Network (TIN) and conversion of TIN to a standard raster. The idea of the linear method is very simple. Any three points, which are not located along a single straight line, may be used to create a plain surface. The values in points located in the triangle of sample points are calculated as elevations of this surface [40].

The next method, Kriging interpolation, has a different nature. While the previous methods are deterministic, this method is stochastic. The Kriging, like other methods of this group, is based on statistical models including autocorrelation of sample points. The basic assumption behind the mechanism of this interpolation is that the distance between sample points is related to the spatial correlation. Then it is used to explain variation in the reconstructed surface. Such an approach enables not only the interpolation but also the assessment of uncertainty. The method is widely used in geology, soil sciences, and other environmental areas. However, the method works well when the spatially correlated distance is known. In many practical cases, it is rather difficult to set the parameters of the Kriging method in the optimal way the [40,45,46,47,48,49,50].

The last method, spline interpolation, is based on the application of cubic polynomials with specific smoothing options to reconstruct curved lines or surfaces. The idea is well known in 1D space and has been extended to 2D surfaces. Even solutions in 1D space with polynomials of a higher order lead to the overestimation or underestimation of the values. The splines are more accurate than the typical polynomial interpolation, but the reconstructed distribution of SPI may be overloaded with errors of this kind [40,50,51,52].

In this study, the data from the period 1990–2020, covering 31 years with 12 months each were analyzed. The five methods were tested, which generated 1860 raster layers. Two approaches were tested, with and without the control station. Hence, the total number of generated interpolated surfaces was 3720. Additional computations, such as processing and preparation of data and analysis of results, were also performed. Because the processed sets of data were rather large, and a huge number of computations were made, the Python language was applied for the automation of this problem. Python is a well-known scripting language [53]. One of its effective areas of application is data analysis including geoprocessing. In our research, the basic Python modules, such as request, zip, cvs, os, shutil, and others, were applied to basic downloading of the data, unzipping, reading, and, in general, pre-processing. However, the most important is a module called ArcPy [54], which is the element of ArcGIS. This Python module enables access to all tools of the ArcToolbox and also enables direct manipulation of the geospatial data.

**Figure 3:**
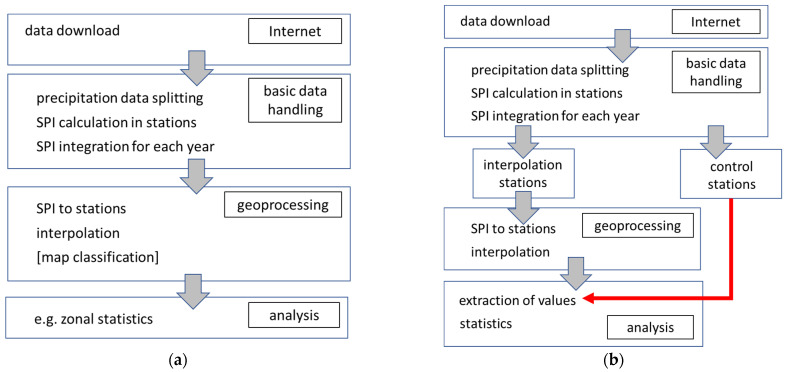
Developed algorithms for interpolation: (**a**) basic form (without control stations), (**b**) advanced form for accuracy assessment (with control stations).

Two algorithms were created (Figure 3a,b). The first was the computational scheme of the basic interpolation (Figure 3a). The data were downloaded in the primary step. Then the precipitation was read from year-by-year files and sorted in a station-like order.

With data pre-processed in such a way, the SPI could be calculated. In this step, finally, the SPI is saved in the year-by-year order to apply for the creation of the SPI maps. The next part includes geoprocessing steps. The SPI is assigned to the proper locations year after year. For each year the interpolation procedure is run. Finally, the maps of SPI may be classified if necessary. Additionally, the zonal statistics or other important information may be drawn from the maps.

**Figure 4:**
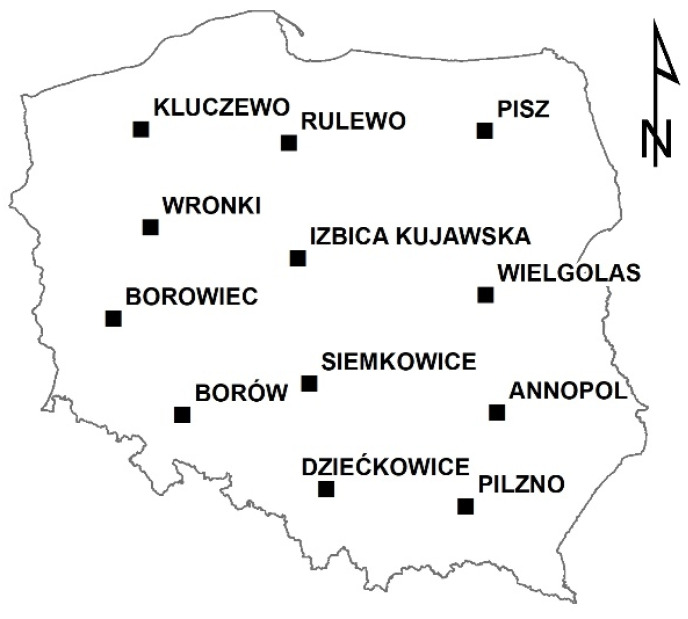
Location of control stations.

The second computational scheme is a little bit different (Figure 3b). Twelve stations are chosen as control stations, as presented in Figure 4. The stations were chosen in such a manner that the distribution of control stations over the territory of the country is more or less uniform. The control stations are removed from the set of interpolation stations before the interpolation method is run. Instead, the control stations are used to extract the interpolated values at their locations and compare them with the values directly calculated. The number of control stations is relatively small because the removal of each station may significantly change the interpolated patterns of SPI, which was noticed during initial computations. This fact is especially important for the accuracy of computations for the eastern part of Poland, where the density of the stations is smaller. The absolute error was chosen as the main measure of inaccuracy.

(3) $$\varepsilon =\left|{\mathrm{SPI}}_{calc}-{\mathrm{SPI}}_{inter}\right|$$

For the sake of simplicity, this basic measure is assumed to describe satisfactorily the accuracy of the applied methods. It is also used as the main reference in the assessment of the interpolation. To quantitatively assess these aspects, the calculated errors were analyzed in a statistical sense as mean error averaged over the years, standard deviations, and the minimum and maximum differences of interpolated and directly calculated values. The proper SPI class identification was also checked.

The entire assessment of accuracy including comparisons of different methods is the basis for the qualitative assessment of uncertainty related to the determination of the drought maps. However, the complexity of the problem is deeper, and more illustrative examples of specific cases are also necessary. Hence, the total assessment of the uncertainty is rather descriptive, though it is supported by analysis of accuracy based on the measurements (3).

## 4. Results

**Figure 5:**
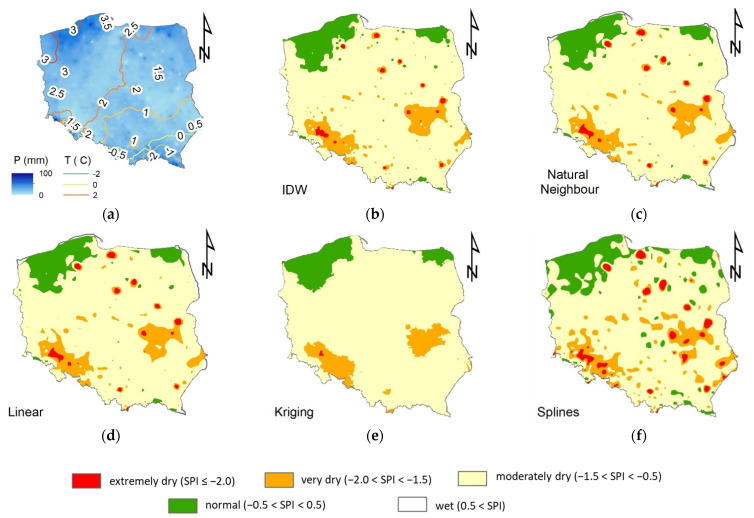
Interpolated monthly results for the beginning of the period of study, January 1990. Part (**a**) presents monthly sums of precipitation and isolines of mean temperatures. The other parts show interpolation of SPI-1 with different methods (**b**) Inverse Distance Weighted, (**c**) Natural Neighbor, (**d**) Linear algorithm, (**e**) Kriging, (**f**) Splines.

The first interesting results were obtained in the basic procedure of the interpolation, without separation of the control stations. An example of such results is presented in Figure 5. There are six maps prepared based on the data from the beginning of the period of study 1990–2020, namely January 1990. The first map (Figure 5a) presents the monthly sum of precipitation. The isolines there represent the average temperatures. The next maps include interpolations of the SPI-1 obtained with Inverse Distance Weighted (Figure 5b), Natural Neighbor (Figure 5c), Linear (Figure 5d), Kriging (Figure 5e), and Spline (Figure 5f), respectively.

All of the methods provided compatible results, as presented on the maps. In the northern part of Poland, there are two zones of normal conditions (−0.5 < SPI < 0.5) when the majority of the country is moderately dry (−1.5 < SPI ≤ −0.5). In the central part, there are two zones of very dry conditions (−2.0 < SPI ≤ −1.5) and, in some cases, small isolated zones of extremely dry conditions (SPI ≤ −2.0). However, the latter is not visible in the Kriging interpolation (Figure 5e). The Spline interpolation gives significantly different results than the other methods. The results presented in Figure 5f are more variable. However, the differences are also clearly visible in other cases. The shapes of zones and the areas covered are different, which may also cause differences in the spatial planning of the drought prevention facilities, such as the location of the reservoirs, etc.

**Figure 6:**
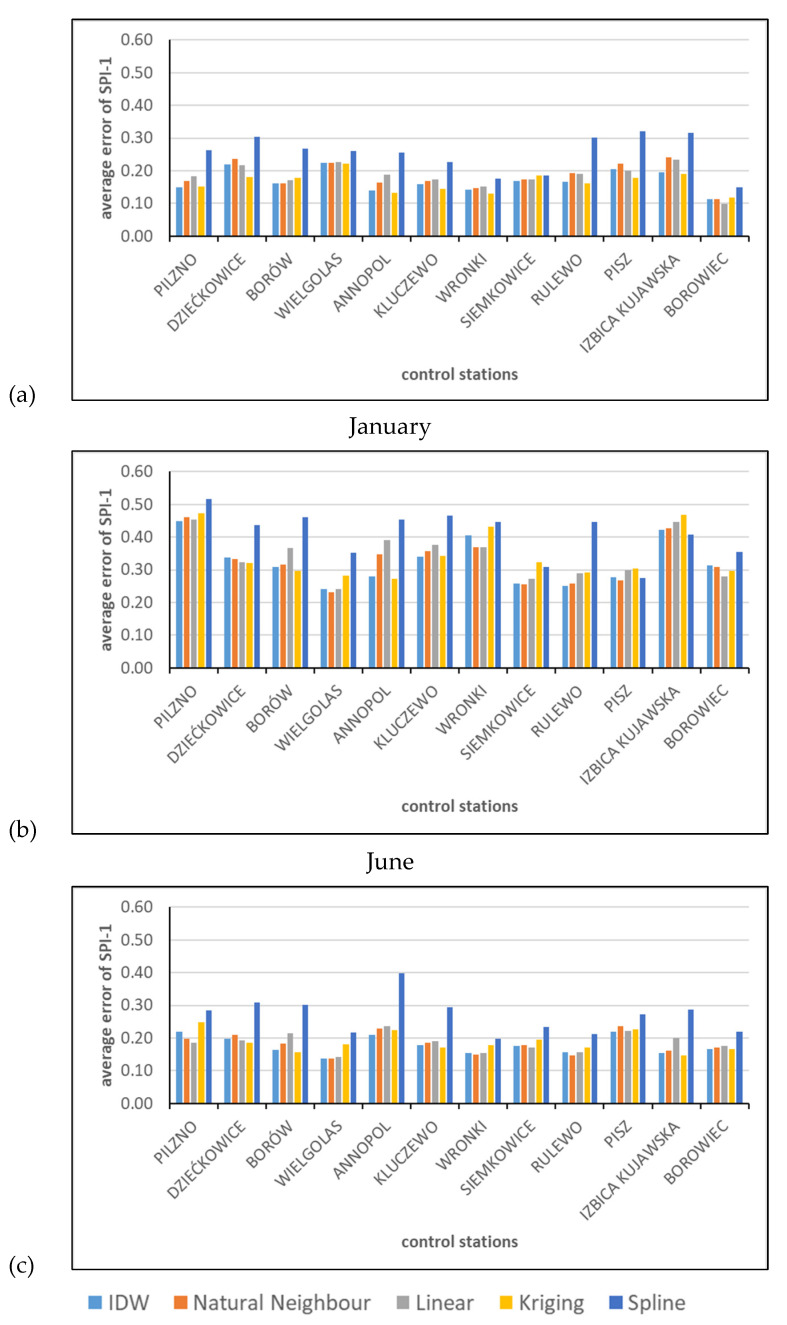
Example of average errors in control stations for (**a**) January, (**b**) June, and (**c**) October.

The next set of exemplary results is presented in Figure 6, showing the average absolute errors between the calculated and interpolated SPI values in the control stations, as found from Equation (3). The 12 control stations are compared, and the period of averaging is 1990–2020. The results for January, June, and October are compared because some monthly patterns related to seasonal variability were noticed. In general, the errors in January were smaller than the errors in June. The results obtained for October were in between the earlier two. The errors in January were in the range of 0.15–0.30, and in June the variability was 0.25–0.50. The more moderate results of October were between 0.15 and 0.40. In all cases, the standard deviations were of the same order as the mean values, which means that sometimes the interpolation was very accurate, but in other cases it may provide values with errors much greater than the average.

In general, the worst results were provided by the Spline interpolation. The best method seems to be the recommended Inverse Distance Weighted. However, Natural Neighbor and Linear interpolations may give similar results. Sometimes, the Kriging method may provide better results, but sometimes worse.

**Figure 7:**
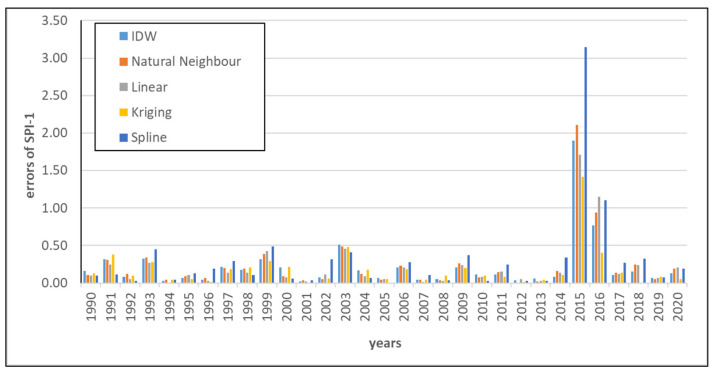
Example of disturbances in control stations, Dziećkowice, month: January, errors for each year of 1990–2020.

**Figure 8:**
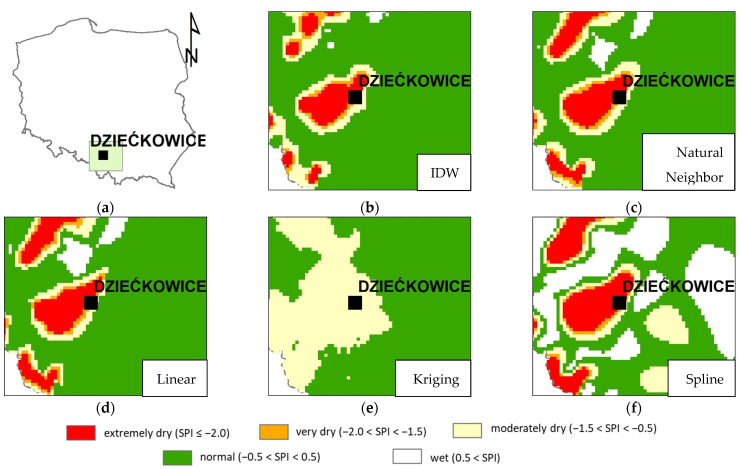
Explanation of disturbances in the measurements at the control station of Dziećkowice, January 2015. Part (**a**) presents localization of control station in Poland. The other parts show interpolation of SPI-1 with different methods: (**b**) Inverse Distance Weighted, (**c**) Natural Neighbor, (**d**) Linear method, (**e**) Kriging, (**f**) Splines.

**Figure 9:**
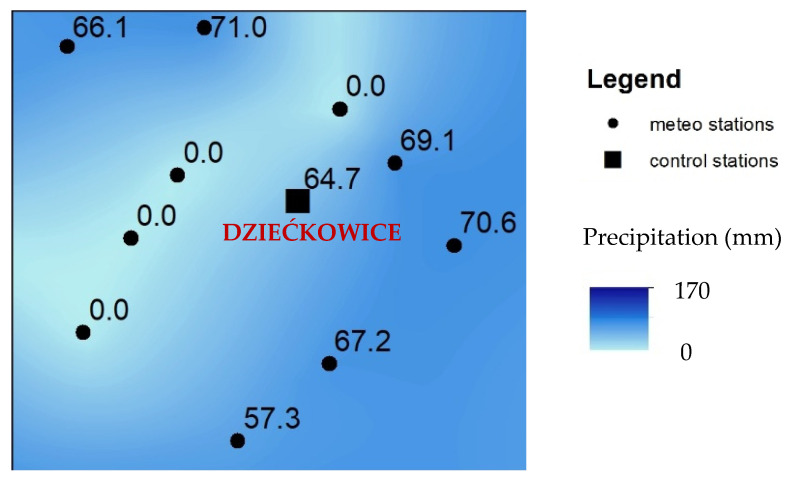
Explanation of disturbances in the measurements at the control station of Dziećkowice, January 2015: numbers are monthly sums of precipitation in control stations.

Sometimes greater errors of interpolation were observed. An example is presented in Figure 7, showing the absolute direct differences between the calculated and interpolated SPI in the control stations located in the southern part of Poland (Figure 8a). The station is called Dziećkowice. The results for January are presented in the graph. In general, the errors are moderate and do not exceed 0.50. However, the results obtained for January 2015 and 2016 show the total failure of all interpolation methods. The explanation of this fact may be found in Figure 8 and Figure 9. In this period, the Dziećkowice control station is located in the area of huge precipitation variability. This phenomenon is presented in Figure 8b–f as rapid changes in the SPI classes obtained with almost all interpolation methods. In Figure 9, the presented control station is located near the border of two different zones of greater precipitation. The four stations with zero precipitation form this invisible border. It is the image of real uncertainties, which are difficult to catch because the location of such areas is not known a priori. It also proves that the control station Dziećkowice is located correctly, just where it should be. If this station was used in the interpolation, the classification would probably be correct.

A summary of the results for the entire period is presented in Table 2, which gives the total mean errors in the interpolation of the SPI values, their standard deviations, and maximum noticed errors. It is well visible that results of the Inverse Distance Weighted method are slightly better than those provided by the Natural Neighbor and Linear methods. 

Unfortunately, the large standard deviations indicate that the errors may be much greater than the presented averages. In general, the variability of the errors is great and the methods should be treated as uncertain. The maximum values presented in the last column substantiate this conclusion.

**Figure 10:**
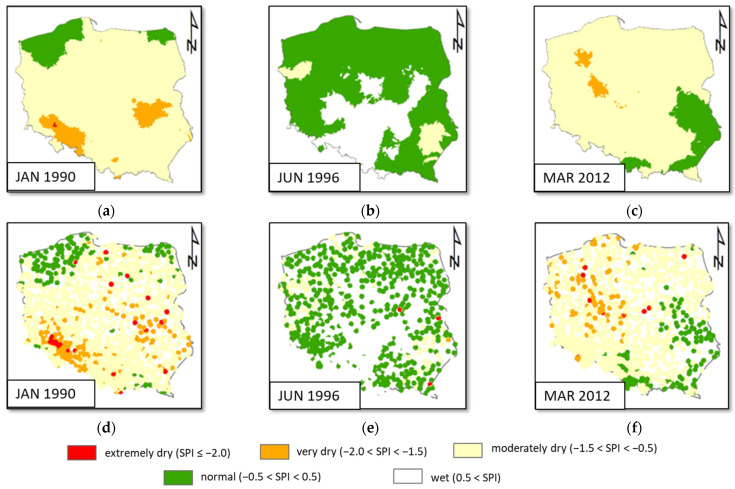
Examples of Kriging method results: in the top row, (**a**–**c**), results with parameters calibrated to obtain interpolation compatible with those of the other methods, in the bottom row, (**d**–**f**) interpolation with default parameters, (**a**,**d**) January 1990, (**b**,**e**) June 1996, (**c**,**f**) March 2012.

The Kriging method seems to give better results than the Linear method and very close results to those brought by the Natural Neighbor and Inverse Distance Weighted methods. The above conclusion is based on the values of the average error as well as the standard deviation. However, the maximum error provided by the Kriging method was greater, which may be related to rather difficult problems with the proper setting of the method parameters. This fact is also illustrated in Figure 10 showing three examples of Kriging interpolation, referring to January 1990, June 1996, and March 2012. In the top row, we may see the maps of interpolated SPI with the parameters of the method set in a way compatible with those of the other methods. At the bottom, there are the results obtained without compatibility. As follows from the data presented, the Kriging method may provide completely wrong results. This method is rather difficult to use properly, which causes real practical problems.

As evidenced by the data shown in Table 2 and the above figure, the Spline interpolation provides the results of the worst quality. Supposedly, the method overestimates and underestimates the interpolated values. This effect is well-known in polynomial interpolation and the specific conditions of the Spline method, which should guarantee the smoothness of interpolation are not able to prevent this problem.

**Figure 11:**
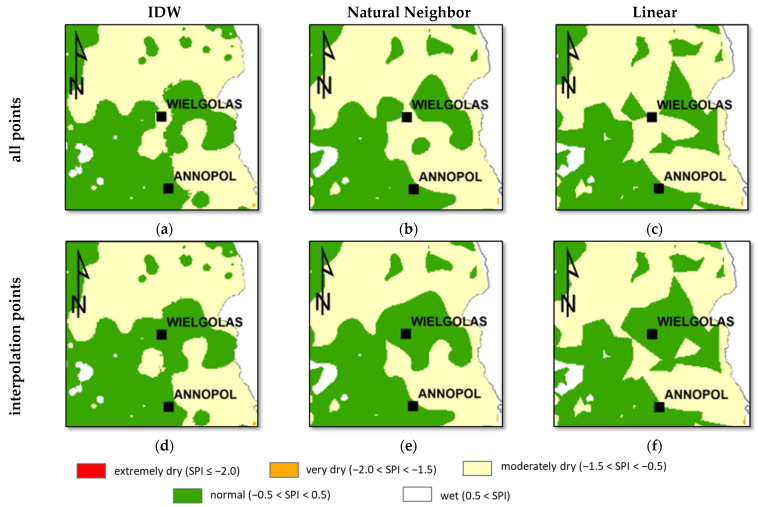
Illustration of sensitivity of interpolation to the point removal. The analyzed period is June 2015. The top row (**a**–**c**), presents the results of the interpolation with all meteorological stations included. The bottom row (**d**–**f**) presents the results without control stations. (**a**,**d**) Inverse Distance Weighted, (**b**,**e**) Natural Neighbor, (**c**,**f**) linear interpolation.

In general, the Inverse Distance Weighted, Natural Neighbor, and Linear methods give similar accuracies at the control stations. All three are sensitive to the presence and removal of the interpolation points. This effect is presented in Figure 11, which concerns the eastern part of Poland with the control stations Wielgolas and Annopol. The period analyzed is June 2015. The top row presents results of interpolation made taking into account all meteorological stations. It means the control stations are not excluded. The bottom row shows the results of interpolation with the data from control stations left out. Wielgolas and Annopol stations are used for assessing the accuracy, and these are not applied in the interpolation process. It is easily seen that the removal of the Wielgolas data significantly changes the classification of the SPI zones. The earlier normal conditions are converted to moderately dry. The effect is visible in the results of all presented methods.

Additionally, it should be noted that the Linear method provides unnatural straight boundaries of the SPI zones. It is typical of this method and only the increase in the control stations density could improve this picture. But this density is rather low in the area analyzed as shown in Figure 2. In the authors’ opinion, the sensitivity to the density of interpolation points is the reason why the linear interpolation methods should not be used in the analyzed case.

Hence, the results prove that all methods are inaccurate and the provided results should be treated as uncertain. Nonetheless, the Inverse Distance Weighted and Natural Neighbor methods seem to be the best suited for the purpose of the study and seem to cause the fewest practical problems with the application to the investigated problem.

## 5. Discussion

Due to high frequency and spatial extent, drought causes environmental and economic losses [55,56,57]. Analysis of the above-mentioned examples emphasizes the importance of monitoring, analysis, and assessment of the impact of drought on global, regional, and local scales. One of the fundamental methods applied for this purpose is reconstruction of a drought’s spatial extent on the basis of drought indices. Usually, drought indices are calculated based on measurement data from field meteorological stations [8]. Collecting data from field measurements provides high-quality data only in single locations. The data are characterized by a point representation of value and do not provide information on the spatial area. According to Wu et al. [58], the available precipitation data are limited in spatial and temporal scale and drought indices calculated from field measurements do not provide information on spatial resolution. Additionally, collecting data from field measurements is a time-consuming and expensive process. Due to the influence of economic factors, the distribution of meteorological stations is random and there might be areas with a lack of measurements [59]. As a result, at the beginning of the analyzed period in 1990 the number of available stations in Poland was over one thousand, while in 2022 the number was six hundred.

In the last years, with the development of GIS and Python applications, computer software has become the most powerful tool for mapping spatial overview changes in the environment [60]. Field measurements from meteorological stations are used to calculate continuous data based on interpolation methods and allow prediction of data in areas where in-situ data are not included. Different interpolation methods were used for mapping drought [61,62,63,64,65]. Subedi et al. [66] used the Inverse Distance Weighting IDW, Kriging, and Spline methods to analyze spatiotemporal changes during a drought in Texas, USA based on SPEI index. The results were based on the data from 47 meteorological stations collected over the years 1980–2013. The dataset included 70% of test data and 30% of validation data. Their analysis has shown that the Kriging method provides good results for dry conditions. In turn, the results based on the IDW method were correct in dry and wet conditions. The Spline method was characterized by high differences between actual measurements and interpolated data. The three techniques to map drought conditions can be ordered according to reliability as IDW > Kriging > Spline. Similar results have been obtained by Rhee et al. [56] for an analysis of drought in North and South Carolina, USA. As follows from a comparison of a few methods based on deterministic and geostatistical interpolations, IDW was the most useful method to assess drought. Ali et al. [67] used SPI to analyze drought in Iran over the years 1979–2008. Their analysis was performed using IDW, Kriging, and Spline methods based on the data from 27 meteorological stations. According to results of drought mapping, the IDW method was the most useful for assessing drought. In turn, Kumar et al. [68] chose the IDW method to analyze drought areas in India over 1951–2019 on the basis of SPI. The correlation between precipitation data and SPI was strong, and the results of mapping show that drought occurred mainly in southwest areas.

In this study, drought areas in Poland were identified on basis of SPI, and the spatial variability was assessed by applying the IDW, Natural Neighbor, Linear, Kriging, and Splines interpolation methods in the ArcGIS software. Comparing values of mean errors for each interpolation method, the IDW method is characterized by the lowest value equal to 0.230. The highest mean error occurs for Spline and Linear methods with values of 0.319 and 0.243. Comparing values of maximum error, IDW and Linear methods are characterized by the lowest values and are equal to 4.761 and 4.530. The results obtained in this study confirm the previous analyses, and the IDW method was selected as the most useful for assessing drought in Poland. In turn, the Spline method interpolation provides the results of the worst quality, e.g., analysis of SPI-1 in January 2015 showed an error equal to 3 while for other methods the maximum was 2.0. Additionally, the results showed that the Linear method provided unnaturally straight boundaries of the SPI zones.

However, the Natural Neighbor method was not taken into account too frequently in the previous studies. As follows from our study, this method may be a quite good alternative to IDW. The previous research was also focused on selected regions, where climate conditions are more or less similar. In our case, the precipitation variability as well as temperature distributions varied significantly. The non-uniform location of meteorological stations in Poland makes the problem of drought map generation more challenging.

A definite advantage of the presented work is providing the applicability of Python scripting for the automation and processing of meteorological data on large spatial and temporal scales. Such an approach enables analysis of the interpolation accuracy for the entire country over 31 years of precipitation variability. As a result, a large number of maps was generated in a relatively short time. The presented approach opens new opportunities, which are mentioned in the conclusions.

## 6. Conclusions

The Standardized Precipitation Index (SPI) was assumed as the proper measure of precipitation scarcity according to the Polish official recommendations as well as according to the international studies presented in the scientific literature. The SPI determined at particular locations at which meteorological data are collected seems to be well suited to the problem of drought assessment. However, the estimation of the SPI spatial distribution is still a challenge. There are no rules for the determination of such a distribution based on physical laws. Hence, the basic interpolation methods implementing different mathematical mechanisms are applied in this area. The assessment of spatial interpolation accuracy is crucial in such cases.

In the presented study, five spatial interpolation methods were tested for the reconstruction of the spatial distribution of SPI values. The one-month version of this index, called SPI-1, was adopted because it is a more variable measure according to the results presented in different literature examples. The study was made for the entire country of Poland, including spatial and temporal variabilities in the available data. The problems of meteorological station distributions and temporality were addressed in the text. The applied methods tested include the Inverse Distance Weighted, Natural Neighbor, Linear, Kriging, and Splines. The preliminary results show the compatibility of the methods, but there are also some significant differences in the spatial extent of the drought. The mentioned differences should be carefully taken into account, because their magnitudes may affect all the studies related to drought prevention, e.g., the design and location of new reservoirs, etc.

The presented results prove that all the tested methods are uncertain, but some of them provide better results. The mechanisms applied for the spatial interpolation differ significantly over all tested procedures and this should be the reason for the observed differences. The worst accuracy was provided by the Splines method. For some practical reasons, the other two methods, namely the Linear and Kriging interpolations, should not be recommended. Hence, the Inverse Distance Weighted as well as Natural Neighbor seem to be well suited for this problem. Considering the current recommendation made by the Polish authorities, such as the National Board for Water Management, the Natural Neighbor method should be treated as a very good equivalent of the more frequently used Inverse Distance Weighted method. In this way the research question formulated in the Introduction has been answered. In the problems of drought mapping, the IDW is better than the majority of the spatial interpolation methods applied broadly in many environmental fields. However, the Natural Neighbor method is quite a good equivalent for this procedure. In some cases, this opportunity may be crucial for environmental studies on the scale of the entire area of Poland. It may be also extended to other countries of a similar scale, resulting in important variability of climatic conditions and non-uniformity in the spatial distribution of meteorological stations.

In the presented research the main challenge was related to the processing of a large number of georeferenced data. To overcome all inconveniences and accelerate the generation of the maps, the unique combination of Python scripting and tools available in the ArcGIS Toolboxes was applied. It enabled effective manipulation of the available data, automatic generation of the drought maps as well as a fast drawing of the statistics for final analysis. Such an approach may have several extensions. First of all, it is expected to permit fast generation of drought maps for the current conditions. On the other hand, it enables determination of spatial statistics in contrast to the local statistics calculated at the site of the meteorological stations. Finally, the application of modern Python may also lead to the implementation of new modules such as multiprocessing, threading, numba, and others. Such approaches may significantly accelerate the overall processing of the precipitation data and drought analyses.

## Figures and Tables

**Table 1:** Classes of SPI according to the recommendation of KZGW [36].

Class	Values	Color
wet	0.5 ≤ SPI	
normal	−0.5 < SPI < 0.5	
moderately dry	−1.5 < SPI ≤ −0.5	
very dry	−2.0 < SPI ≤ −1.5	
extremely dry	SPI ≤ −2.0	

**Table 2:** General summary of results for all control stations in all months of the period 1990–2020.

Interpolation Method	Mean Error	STD	Max Error
Inverse Distance Weighted	0.230	0.241	4.761
Natural Neighbor	0.236	0.253	4.755
Linear	0.243	0.259	4.530
Kriging	0.236	0.240	4.804
Spline	0.319	0.371	6.340

## Data Availability

Data sharing is not applicable to this article.
